# Drug Design—Past, Present, Future

**DOI:** 10.3390/molecules27051496

**Published:** 2022-02-23

**Authors:** Irini Doytchinova

**Affiliations:** Drug Design and Bioinformatics Lab, Faculty of Pharmacy, Medical University of Sofia, 1000 Sofia, Bulgaria; idoytchinova@pharmfac.mu-sofia.bg

**Keywords:** drug design, drug discovery and development, QSAR, molecular docking, molecular dynamics, virtual screening, artificial intelligence

## Abstract

Drug design is a complex pharmaceutical science with a long history. Many achievements have been made in the field of drug design since the end of 19th century, when Emil Fisher suggested that the drug–receptor interaction resembles the key and lock interplay. Gradually, drug design has been transformed into a coherent and well-organized science with a solid theoretical background and practical applications. Now, drug design is the most advanced approach for drug discovery. It utilizes the innovations in science and technology and includes them in its wide-ranging arsenal of methods and tools in order to achieve the main goal: discovery of effective, specific, non-toxic, safe and well-tolerated drugs. Drug design is one of the most intensively developing modern sciences and its progress is accelerated by the implication of artificial intelligence. The present review aims to capture some of the most important milestones in the development of drug design, to outline some of the most used current methods and to sketch the future perspective according to the author’s point of view. Without pretending to cover fully the wide range of drug design topics, the review introduces the reader to the content of *Molecules’* Special Issue “Drug Design—Science and Practice”.

## 1. Introduction

A drug is a foreign molecule that affects biological processes and is used to prevent, diagnose or treat a disease [1]. Drugs can be of natural origin or produced synthetically. The ideal drug should have a specific action, be safe, non-toxic, without side effects or as few as possible, be chemically and metabolically stable, be synthetically feasible, be soluble in water in therapeutic concentrations in order to avoid precipitation in the blood stream, be soluble in lipids as well, in order to be able to cross the lipid membranes and distribute around the body, and finally, be a unique molecule [2,3].

In order to exert their effects, drugs interact with specific targets in the human body. As a result of these interactions, two types of effects are produced: effects of the drug on the human body, and effects of the human body on the drug. These effects are considered by Pharmacodynamics and Pharmacokinetics, respectively. [4]. Pharmacodynamics focuses on the mechanisms of drug action, the relationships between drug concentration and effect, and the adverse reactions. Pharmacokinetics studies the absorption, distribution, metabolism and excretion of the drug over time, the so-called ADME processes or ADME properties of drugs.

The process of drug discovery and development consists of three main stages: drug discovery, preclinical development and clinical trials. The drug discovery starts with the finding of a hit molecule. A hit is a molecule that elicits a desired activity in a screening assay [5,6]. Then, the structure of this molecule is optimized in terms of improving affinity and selectivity, reducing toxicity, improving water and lipid solubility, improving ADME properties in general and converting the hit molecule into a lead molecule. The further optimization of the lead molecule delivers the drug candidate. Next, the preclinical studies are focused on clarifying the mode of action of the drug candidate, its pharmacokinetics in animals such as bioavailability, toxic metabolites, if any, routes of excretion, efficacy on animals, drug formulation and stability tests of this formulation. The clinical trials are the longest and the most expensive stage of the process, consisting of three phases. In the first phase, up to 100 healthy volunteers are involved. The aim of this phase is to evaluate the safety of the drug on human, its pharmacokinetics in the human body and immediate side effects if there are any. In the second phase, the drug is administered to several hundred patients suffering from the target disease. At this phase, the efficacy of the drug and its short-term safety are tested. In the third phase, several thousand patients from several clinical centres around the world are involved. The aim of this phase is to collect sufficient data for the efficacy and safety of the drug. If the drug passes this phase successfully, it is ready for registration and marketing.

However, the surveillance of the drug does not stop here. The drug continues to be observed for safety and side effects. This last phase is known as a post-marketing surveillance and it is practically endless, it continues until the drug is on the market.

## 2. Drugs Are High Value-Added Products

**Figure 1:**
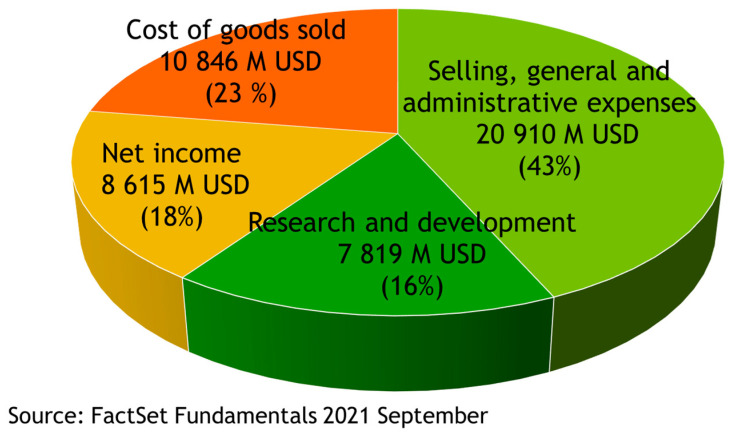
Average costs for five Big Pharma companies (Pfizer, GSK, Roche, Sanofi and Novartis) over the last 10 years, according to their financial reports published in FactSet fundamentals [7].

The pharmaceutical industry is one of the most successful businesses in the world. It is affected neither by financial crises nor by political ones, because sick people always exist, and unfortunately, their number increases during crises. If we look at the financial reports of 5 of the top 10 Big Pharma companies (Pfizer, GSK, Roche, Sanofi and Novartis) for the last 10 years, we can see several quite interesting facts (Figure 1) [7]. The cost of goods sold is only 23% of the total income of the companies, on average. Almost half of the income (43%) is spent on selling, general and administrative expenses. In total, 16% of the total income is reinvested in research and development, which is significantly higher than the average value of 7% for other businesses. Thus, drugs are definitely high-value-added products. The net income is 18%. This puts the pharma industry in the top three most profitable industries in the world.

The cost of developing a drug increases exponentially—it doubles every 10 years. It has been estimated that the cost of a new drug is around USD 2.6 billion in average (2013) [8]. The cost of biologics is especially high—proteins, monoclonal antibodies, diagnostic products and vaccines [9]. 

Why the process of drug discovery and development is so expensive? One of the possible answers is because of its low efficiency. From ten thousand synthesised and tested compounds, around 100 show some activity and safety, 10 of them enter the clinical trials and only 1 is approved for medical use [2]. Recent studies show that the initial number of tested compounds even passes one million [10]. Additionally, this process takes up to 12 years. However, as with the COVID-19 vaccines last year, we have seen that the time for drug development can be significantly reduced. 

Drug discovery and development is a high-risk business. In average, 7 out of 10 projects are cancelled preliminary because of different reasons [11]. The main reason is the lack of efficacy, i.e., the drug is effective on animals but when administered to humans the therapeutic effect is absent or is negligibly small [12]. The second main reason in the past was the pharmacokinetics of the new drug—low bioavailability, toxic metabolites, short or extremely long half-lives. However, during the last 20 years many in silico tools and models have been developed to assess the physicochemical and ADME properties of drug candidates during the experimental stage and the attrition rate due to decreases in pharmacokinetics from 39% in the past [13] to the current negligible 1% [11]. Animal toxicity, adverse reactions, commercial and other issues are among the other attrition reasons.

Based on the reasons described above, it is obvious that drugs are high-value-added products and the new drugs are highly expensive. Unfortunately, even nowadays drugs are non-affordable products for most people on this planet. 

## 3. Drug Discovery

There are different approaches for drug discovery. The oldest one is by serendipity. Serendipity means discovery by chance—trial and error. There are many examples in the history of pharmacy for drugs discovered by serendipity [14], starting with the most popular—the story about penicillin, a drug which saved millions of lives during the Second World War and for which Fleming, Florey and Chain received the Nobel Prize in 1945. This drug is still in use. Chlordiazepoxide, the first benzodiazepine, was also discovered by serendipity. In the 1930s, Leo Sternbach from the University in Cracow synthesized several heptoxdiazines in order to develop synthetic dyes. Cyclosporin was testing as an anti-tubercular antibiotic but became the first immunosuppressive drug that changed the science and practice of organ transplantation [15]. Additionally, the latest story is about sildenafil (Viagra), which was developing as an antihypertensive drug but is becoming one of the best-selling drugs ever, leading to an entirely new pharmacological group in modern pharmacology.

Another approach for drug discovery is by chemical modifications of known drugs or natural products [2,16]. Aspirin was discovered by chemical modification [17]. The natural product salicylic acid was acetylated in order to increase the stability and reduce the irritating effect on stomach mucosa. Small chemical modifications lead to improved therapeutic profiles in drugs of different generations. For example, ranitidine is a chemical modification of cimetidine with higher potency and prolonged half-life [18], pindolol originates from propranolol but avoids the first-pass effect in the liver and shows a higher degree of bioavailability [19].

Screening of databases, virtually or by high throughput (HTS) assays, is another way to discover new drugs [20,21,22]. The first sulphonamide drug Prontosil was discovered by random in vitro screening, when a great number of colorants were screened for antibacterial activity [2,23]. Paclitaxel, a novel anti-tumour agent, was discovered by HTS as well [24]. 

Nowadays, the most advanced method for drug discovery is the rational drug design [3,25]. This is the smartest and the cheapest approach of drug discovery. Drug design begins with an identification of a biological target (a biomacromolecule involved in the disease). Then, a ligand interacting with this macromolecule, known as a hit molecule, has to be discovered. It follows an iterative process of structure optimization until a compound is derived with optimal affinity, selectivity, non-toxicity, solubility, permeability, bioavailability, etc., properties which are needed for a molecule to become a drug [3]. There are two main approaches in drug design: ligand-based and structure-based [26]. When the structure of the target macromolecule is unknown, the structure of the ligand is designed and optimised based on the relationship between structure and activity. In the structure-based drug design, the 3D structure of the target macromolecule is known and the ligand is designed to be complementary to the binding site on the macromolecule. Complementarity means a steric, electrostatic and hydrophobic fit between the ligand and the target. 

## 4. Drug Design—Historical Notes

There were several major achievements in the science of drug design that made it the main approach in the current and the future drug discovery [2]. The first of them is the understanding of drug–receptor recognition. In the early 1890s, Emil Fisher compared the drug–receptor interaction to the key and lock interplay. He considered that both the drug and the receptor interact as solid bodies without changing their conformations. Lately, Daniel Koshland suggested that both molecules do undergo conformational changes during interaction and adopt the most suitable conformation in order to connect each other. This hypothesis has been proven many times by X-ray structures and in silico simulations and now it is known that ligands indeed change their conformations during the interaction and adopt conformations that optimally fit the contact surfaces. 

**Figure 2:**
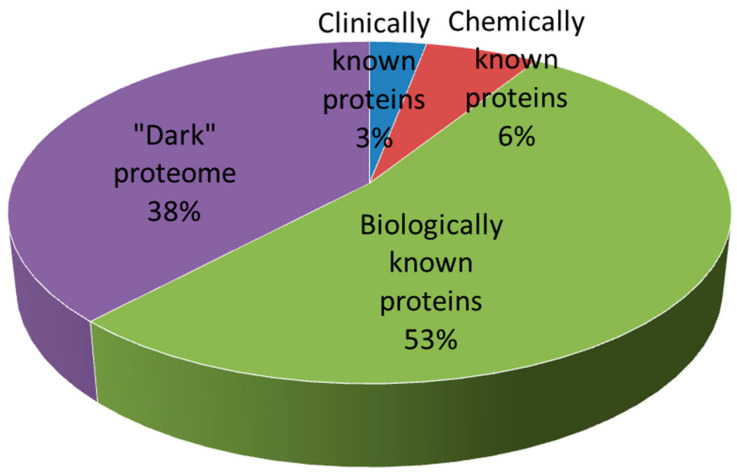
The human druggable proteome divided according to the target development level [28]. Clinically known targets includes proteins linked to at least one approved drug. Chemically known targets includes proteins known to bind with high potency small molecules that are not yet drugs. Biologically known targets refer to proteins that have a link to any disease but have not been studied for binding to small molecules. The “dark” proteome includes the unstudied proteins.

The target macromolecule is an internal molecule which is involved in the disease. Affecting the functions of target macromolecule could change the etiology of the disease, the pathophysiology or simply improve the symptoms. Of the 20,000 protein-coding genes in the human genome, about 3 000 have been estimated to be part of the so-called druggable genome or druggable proteins [27]. These are proteins that have the ability to bind drug-like molecules. Oprea et al. [28] divided the whole proteome into four groups according to the target development level, i.e., how much we know about a given protein (Figure 2). Clinically known targets include macromolecules linked to at least one approved drug. Theirnumber is 659, i.e., 3% of the human proteome. Of them, 25% are enzymes, 21% are ion channels, 16% are gamma-protein-coupled receptors, 9% are different types of kinases, 4% are transporter proteins, 3% are nuclear receptors and the remaining 22% include other protein families and orphan receptors. Chemically known targets include proteins known to bind with high potency small molecules that are not yet drugs. They constitute 6% of the human proteome. Biologically known targets refer to proteins that have a link to any disease but have not been studied for binding to small molecules. More than half of the human proteome (53%) belongs here. The “dark” targets include the unstudied proteins. They are 38% of the whole proteome. Obviously, there is a huge field for future discoveries. Since 2014, there has been an initiative funded by the NIH aiming to illuminate the druggable genome. All new data discovered are collected on the Pharos website (pharos.nih.gov, accessed on 20 February 2022) [29].

The 3D structures of proteins are resolved by X-ray crystallography, NMR spectroscopy and more recently by the modern cryogenic electron microscopy, and collected in the Protein Data Bank (PDB). PDB currently contains more than 180 thousand structures [30]. Most of them are single proteins, in apo-form, without a ligand. However, some of them are in complexes with ligands and this information is more valuable. It locates the protein-binding site.

The major achievement in the modern drug design is the development of in silico modelling technologies, applied for virtual screening, compound design, energy calculations, SAR and QSAR analysis, ADME modelling, modelling of drug–target interactions. In order to be applied all these advanced technologies, the molecular structure should be encoded numerically [31]. The encoded structures can be analysed, searched, visualized and compared with other structures. The structures could be encoded by binary strings, smiles strings, 2D graphs and 3D structures. The 3D molecular modelling and visualisation also are considered among the greatest achievements in drug design technologies. 

The molecular properties also should be encoded. Different types of descriptors have been developed over the years. The 1D descriptors are derived from the 1D structure such as element composition and molecular weight. The 2D descriptors are generated from the molecular graph such as the number of hydrogen bond acceptors and donors, the partition coefficients logP and logD, water solubility, etc. The 3D descriptors are extracted from the 3D structures. These are molecular surface area or polar surface area, molecular volume, etc. Carracedo-Reboredo et al. define 4D descriptors, providing information about the interactions between ligands and protein-binding sites [32]. Apart from single numbers, descriptors could be composed of several numbers. These are the descriptor sets which describe a complex property or a set of properties.

Once there is a quantitative description of a set of structures and a quantitative measurement of their activities such as IC_50_, EC_50_, K_d_, etc., then different types of quantitative analyses can be applied. Corwin Hansch conducted this for the first time in 1964 when correlating the antimicrobial activity of penicillin derivatives with descriptors relating to the hydrophobic and electronic properties of the molecules [33]. The correlations between molecular descriptors of a series of compounds and their activities are assigned as Quantitative Structure Activity Relationships (QSAR). Corwin Hansch (1918–2011) is considered the father of drug design [34]. 

Lately, in the analyses have been involved methods such as principal component analysis [35] and partial least squares [36]. Chemometrics evolved into chemoinformatics including molecular modelling, chemical information and tools and algorithms to handle the enormous amount of data coming from the combinatorial chemistry and HTS [37]. At the same time, bioinformatics also emerged to organise and analyse biological data, the amount of which increased dramatically, especially after deciphering the human genome. Nowadays, the structure–activity relationships are analysed by machine learning methods such as random forest, decision trees, neural networks, k nearest neighbours, etc. [38,39]. Many authors classify these methods as “black box” methods because they are not able to distinguish immediately between relevant and irrelevant for the activity descriptors as the regression methods do [39]. However, they are very good in predictions and classifications, significantly better than the statistical analyses and regression models. Both types of methods do not replace each other, nor contradict. They are complimentary and most effective when are used in combination.

## 5. Current Methods for Drug Design

Currently, artificial intelligence (AI) has invaded drug discovery in all aspects of this process [40,41,42]. In drug design, AI is used to predict the 3D structure of proteins, drug–protein interactions and drug activity, constructs molecules de novo. In pharmacology, AI is used to design specific molecules as well as multitarget drugs. In chemical synthesis, AI is able to design synthetic route, to predict reaction yield, to clarify reaction mechanisms. AI is quite good at repurposing old drugs to new therapeutic targets. Undoubtedly, AI is irreplaceable in drug screening for predicting toxicity, bioactivity, ADME properties, physicochemical properties, etc.

Among the most popular AI platforms used in drug design is the SwissDrugDesign system developed by the Swiss Institute of Bioinformatics [43]. The platform is freely accessible via the Expasy portal (https://www.expasy.org, accessed on 20 February 2022) and consists of several modules: molecular docking (SwissDock) [44], pharmacokinetics and drug likeness prediction (SwissADME) [45], virtual screening (SwissSimilarity) [46], lead optimisation (SwissBioisostere) [47] and target prediction of small molecules (SwissTargetPrediction) [48]. 

AlphaFold is the first computational method for predicting the 3D protein structute based solely on its amino acid sequence developed by DeepMind and EMBL-EBI [49]. AlphaFold incorporates a neural network architecture trained on PDB by implicating evolutionary, physical and geometric constraints of protein structures. A recent CASP14 assessment shows that AlphaFold demostrates the most accurate prediction of 3D protein structures with median backbone RMSD = 0.96 Å [49]. 

Merck’s Synthia (enhanced Chematica) proposes possible synthesis routes for a given compound. The AI tool allows by customizing the search parameters such as rules, filters, scoring functions and stop conditions to generate several synthetic pathways for a molecule of interest. Klucznik et al. used Chematica to design the synthetic routes of 8 known compounds and then experimentally tested them. Yield improvements and cost saving over previous approaches have been observed for all compounds [50]. 

Cyclica’s Ligand Express finds possible protein targets for a given small molecule. Opposite to screening a database of small molecules to find the proper ligand for a given protein, this cloud-based platform screens the human proteome in order to find the proper target protein or proteins for a given small molecule [51]. 

AstraZeneca’s AI platform for de novo design of small molecules, named REINVENT is able to generate small molecules that satisfy a diverse set of criteria defined by the use [52]. It is an open-source Python application and the code is freely available at https://github.com/MolecularAI/Reinvent (accessed on 20 February 2022) for downloading.

Many other AI tools and platforms for drug discovery and development are available in the web and new ones are inconstantly appearing. The detailed presentation of all of them is out of the scope of the current review but they are analysed and compared in several good reviews [40,41,42,52,53,54,55]. 

The data visualization is of great importance for drug design. Here, among the great achievements are the techniques of molecular dynamics and molecular docking. Both of them were possible because of the invention of molecular mechanics [56]. The molecular mechanics is based on a set of empirical energy functions called a force field. The force field is able to compute the conformational energy of the system, consisting of several terms such as bond stretch energy term, bond angle energy term, torsional energy, van der Waals interactions, intramolecular as well as intermolecular interactions, electrostatic interactions and hydrogen bond formation [57].

Molecular dynamics (MD) is a method for simulating the movements of molecules and their interactions in different media based on the force field [58]. MD provides information that cannot be obtained by any experimental method for 3D structure resolution such as crystallography, spectroscopy and microscopy. These methods give a snapshot of the ligand-protein complex, whereas MD mimics the movements of the molecules and their interactions over time. 

Molecular docking predicts the mode of binding between two molecules, the mutual orientation of the molecules, the conformation of each molecule and estimates the energy of the complex. The lower is the energy, the more stable the complex. Through docking-based virtual screening, hit structures can be identified among huge datasets binding to a given binding site [20]. Another type of virtual screening is pharmacophore-based screening [59]. The pharmacophore is a 3D ensample of functional groups in the active molecules necessary for binding to a given receptor. For the best performance, both approaches for virtual screening are complimentarily used [59,60].

The cherry-top of the automation and current application of artificial intelligence in drug design are the robots Adam and Eve, constructed by Ross King and Stephen Oliver from the University of Manchester. Adam was constructed to run microbiological experiments, analyse the results itself, define hypotheses, design experiments to test these hypotheses and repeat this cycle until a validated hypothesis is derived [61]. Eve is a more advanced robot. She is able to screen experimentally thousands of compounds per day, to discover specific hits, to engineer a specific cell line to test the hits and then to optimize their structures to deliver lead compounds [62].

## 6. Future Trends in Drug Design

Any advance in science and technology finds immediately its application in medicine, in pharmacy, in drug discovery and development. Investments in drug design are worthwhile because as better is designed a given drug candidate during the experimental stage, as less likely is for the drug to fail in the late stages where the tests are more expensive, especially in the clinical trials. The COVID pandemic forced us to rethink how to accelerate the timelines of discovery and development of drugs and vaccines. New, effective, and less costly methods for drug discovery are required and AI has the potential to provide them. AI is able to gather and analyse large amounts of data in a short time, to select appropriate targets and complimentary ligands, to design tests and to perform them. The ultimate goal of the future drug design is to be able to design and develop a specific, non-toxic, effective and patient-tailored drug over a period of several hours. Although this goal seems fantastic at the moment, it is completely achievable in the near future.
